# Genetically stratified Parkinson’s disease with freezing of gait is related to specific pattern of cognitive impairment and non-motor dominant endophenotype

**DOI:** 10.3389/fnagi.2024.1479572

**Published:** 2024-10-11

**Authors:** Lukas Pavelka, Rajesh Rawal, Stefano Sapienza, Jochen Klucken, Claire Pauly, Venkata Satagopam, Rejko Krüger

**Affiliations:** ^1^Translational Neuroscience, Luxembourg Centre for Systems Biomedicine (LCSB), University of Luxembourg, Esch-sur-Alzette, Luxembourg; ^2^Transversal Translational Medicine, Luxembourg Institute of Health (LIH), Strassen, Luxembourg; ^3^Parkinson’s Research Clinic, Centre Hospitalier de Luxembourg (CHL), Luxembourg, Luxembourg; ^4^Biomedical Data Science Group, Luxembourg Centre for Systems Biomedicine (LCSB), University of Luxembourg, Esch-sur-Alzette, Luxembourg; ^5^Digital Medicine, Luxembourg Centre for Systems Biomedicine (LCSB), University of Luxembourg, Esch-sur-Alzette, Luxembourg; ^6^Digital Medicine, Department of Precision Health, Luxembourg Institute of Health (LIH), Strassen, Luxembourg

**Keywords:** Parkinson’s disease, non-motor symptoms, cognitive subdomain, executive dysfunction, visuospatial impairment, endophenotype

## Abstract

**Background:**

Freezing of gait (FOG) is an important milestone in the individual disease trajectory of people with Parkinson’s disease (PD). Based on the *cognitive model* of FOG etiology, the mechanism behind FOG implies higher executive dysfunction in PD^FOG+^. To test this model, we investigated the FOG-related phenotype and cognitive subdomains in idiopathic PD (iPD) patients without genetic variants linked to PD from the Luxembourg Parkinson’s study.

**Methods:**

A cross-sectional analysis comparing iPD^FOG+^ (*n* = 118) and iPD^FOG−^ (*n* = 378) individuals was performed, followed by the application of logistic regression models. Consequently, regression models were fitted for a subset of iPD^FOG+^ (*n* = 35) vs. iPD^FOG−^ (*n* = 126), utilizing a detailed neuropsychological battery to assess the association between FOG and cognitive subdomains. Both regression models were adjusted for sociodemographic confounders and disease severity.

**Results:**

iPD^FOG+^ individuals presented with more motor complications (MDS-UPDRS IV) compared to iPD^FOG-^ individuals. Moreover, iPD^FOG+^ individuals exhibited a higher non-motor burden, including a higher frequency of hallucinations, higher MDS-UPDRS I scores, and more pronounced autonomic dysfunction as measured by the SCOPA-AUT. In addition, iPD^FOG+^ individuals showed lower sleep quality along with lower quality of life (measured by PDSS and PDQ-39, respectively). The cognitive subdomain analysis in iPD^FOG+^ vs. iPD^FOG−^ indicated lower scores in Benton’s Judgment of Line Orientation test and CERAD word recognition, reflecting higher impairment in visuospatial, executive function, and memory encoding.

**Conclusion:**

We determined a significant association between FOG and a clinical endophenotype of PD with higher non-motor burden. While our results supported the cognitive model of FOG, our findings point to a more widespread cortical impairment across cognitive subdomains beyond the executive domain in PD^FOG+^ with additional higher impairment in visuospatial function and memory encoding.

## Background

Parkinson’s disease (PD) is one of the most intriguing chronic neurodegenerative disorders steadily on the rise in terms of prevalence and incidence, significantly contributing to an overall disease burden of neurological disorders in the world (Steinmetz et al., 2024). It is estimated that from 1990 to 2040, the overall prevalence of PD will show a dramatic seven-fold increase, a phenomenon that cannot be solely attributed to the aging population or better diagnostic measures (Dorsey et al., 2018). In addition, cumulative evidence indicates that the pathological processes associated with PD can start many years (even up to 20 years) before the appearance of the first cardinal symptoms (Mahlknecht et al., 2015), which include bradykinesia, extrapyramidal rigidity, and/or resting tremor. During the course of the disease, additional motor symptoms emerge, such as gait disorder, falls, dystonia, and freezing of gait (FOG). Nevertheless, gait control is a complex phenomenon depending on a vast number of neural integrators ranging (and not exclusively) from spinal central pattern generators to mesencephalic and cerebellar locomotor areas, subthalamic locomotor regions, and cortical areas such as primary and supplementary motor areas (Weiss et al., 2020).

Freezing of gait is among the most difficult to treat motor complications in PD and significantly increases the risk of falls and related complications (Okuma et al., 2018; Giladi and Nieuwboer, 2008). This loss of gait automation and gait patterning has been studied extensively; however, a comprehensive explanation of FOG mechanism(s) is still lacking (Diederich et al., 2020). In total, four models addressing the multifaceted etiology of FOG have been proposed (Nieuwboer and Giladi, 2013): threshold model, decoupling model, interference model, and cognitive model. Based on the cognitive model of FOG etiology (Vandenbossche et al., 2012), the mechanism behind FOG implies a conflict-resolution deficit in controlling action selection and response inhibition, mainly as a consequence of higher executive dysfunction in PD^FOG+^. Previously, cognitive impairment was considered to be an independent risk for developing FOG and might contribute to the FOG etiology as proposed in the cognitive model of FOG (Kim et al., 2019; Kim et al., 2018). In addition, dual-tasking, external sensory factors, and affective factors (e.g., fear and anxiety) have been shown to trigger or aggravate FOG. This suggests that higher-level cortical modulators play an important role in integrating motor, sensory, and limbic inputs for physiological gait generation (Wu et al., 2015; Hallett, 2008; Heremans et al., 2013).

However, the causality or bi-directional link between FOG and cognition (i.e., cognitive impairment contributing to FOG and/or FOG exacerbates the cognitive impairment) remains unresolved. To address this issue, previous studies yielded highly variable results, mainly due to low overlap in study setups and insufficient intergroup matching for confounding factors such as dopaminergic medication and disease severity. Additionally, many studies did not account for comorbidities that could influence the outcomes and often disregarded the genetic status of PD patients, which may be an independent factor linked to both FOG and cognitive impairment. Specifically, the most common genetic risk factors for PD, mutations in *GBA1*, were reported to be associated with a higher frequency of FOG and cognitive impairment in PD (Yang et al., 2023), as well as a more severe disease progression rate in the longitudinal follow-up (Brockmann et al., 2015).

While addressing the above limitations, we tested the hypothesis behind the cognitive model of FOG and investigated the cognitive performance and cognitive subdomains in iPD without genetic variants linked to PD using a large neuropsychological assessment battery in iPD^FOG+^ compared to iPD^FOG−^. In addition, we enquired whether FOG is associated with a specific clinical endophenotype both in terms of non-motor and motor complications in individuals with iPD.

## Materials and methods

### Study population and ethical considerations

**Figure 1:**
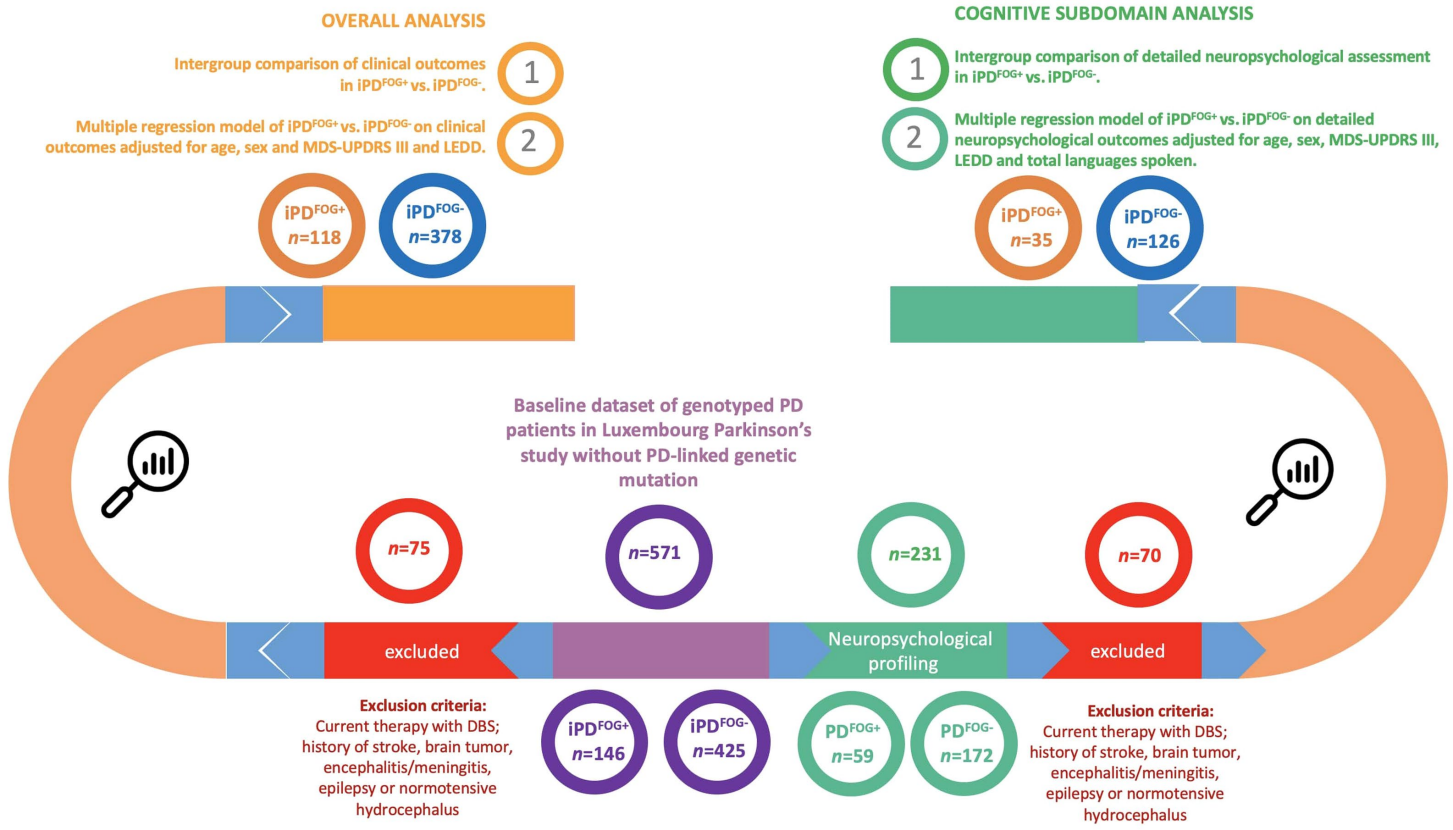
Flowchart describing the selection of individuals and the study design. *Genotyped by NeuroChip (Illumina) and targeted re-sequencing of the *GBA1* gene by PacBio. FOG, Freezing of gait; iPD, Idiopathic Parkinson’s disease; DBS, Deep brain stimulation; MDS-UPDRS, Movement Disorder Society-Unified Parkinson’s Disease Rating Scale; LEDD, Levodopa equivalent daily dose.

The diagnosis of PD was compliant with the diagnostic criteria defined by the United Kingdom Parkinson’s Disease Society Brain Bank (UKPDSBB) (Litvan et al., 2003). The patients were selected from the baseline visit dataset recruited between March 2015 and November 2022 in the Luxembourg Parkinson’s study with available genotyping and history and/or current motor symptoms of FOG. Out of this group, individuals with an available extensive cognitive assessment were included in a subset analysis of cognitive subdomains. The details of the inclusion and exclusion steps are shown in Figure 1.

### Data collection

**Figure 2:**
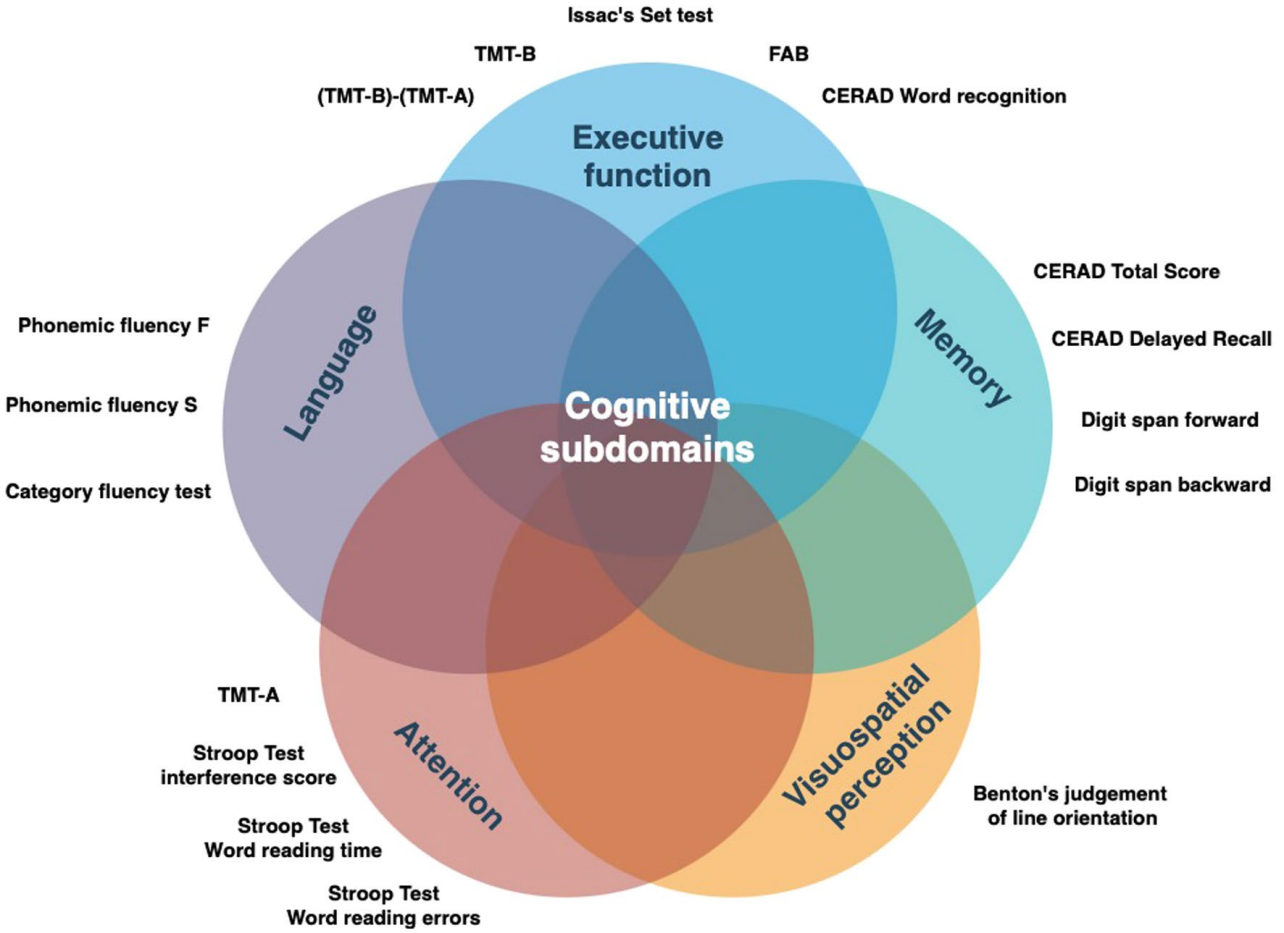
Detailed neuropsychological battery applied in the study categorized by the target cognitive subdomain. FAB, Frontal assessment battery; TMT, Trail making test; CERAD, Consortium to Establish a Registry for Alzheimer’s Disease.

The clinical assessment and baseline characteristics of the Luxembourg Parkinson’s study were previously published (Hipp et al., 2018; Pavelka et al., 2023). The assessment of cognitive subdomains belonged to an optional participation level in the study and was performed by a neuropsychologist. The neuropsychological assessment battery for cognitive profiling is illustrated in Figure 2. Both clinical scales and neuropsychological testing were performed in the medication ON state. Group assignment (PD^FOG+^/PD^FOG−^) was based on the history and/or presence of recurrent FOG events assessed during a semi-structured interview by a study physician. Given the relative rarity of ON-related FOG in comparison with OFF-related FOG, the stratification of FOG to OFF-related vs. ON-related FOG was not performed. The reported clinical scales were validated for use in PD patients and were described in detail previously (Pavelka et al., 2023). The clinical examination and comorbidities were captured as a part of the semi-structured interview between the patient and/or patient’s proxy and study physician. Data export from the electronic database REDCap (Harris et al., 2019; Harris et al., 2009) (baseline visit) was performed on 22nd November 2022.

### Genotyping and quality-control analyses

Genotyping comprised screening via NeuroChip (Illumina) and targeted re-sequencing of the *GBA1* gene by PacBio, as previously described in detail (Pachchek et al., 2023; Pavelka et al., 2022). PD-causing rare variants were defined using the ClinVar classification as “pathogenic/likely pathogenic” and were used as exclusion criteria for the cross-sectional analysis. The pathogenic variants used for exclusion were listed in Supplementary material.

### Missing data statement

The absolute number and proportion of missing values per variable are described in Tables 1, 2. Given the overall low proportions of missing values in the dataset, we used a pairwise deletion for all statistical models.

**Table 1:** Sociodemographic and clinical profile of patients with idiopathic Parkinson’s disease (iPD) from the Luxembourg Parkinson’s study with and without freezing of gait (FOG).

		iPD^FOG−^	iPD^FOG+^	iPD^FOG+^ vs. iPD^FOG−^	
Clinical and demographic variables	Missing	Mean (SD) or YES (%)	Mean (SD) or YES (%)	OR [CI 95%]	*p* value
	*n* (%)				
Number of individuals (*n*)		378	118	-	-
Sex (male)^a^	0 (0)	241 (63.8%)	88 (74.6%)	1.66 [1.05;2.68]	0.868
Family history of parkinsonism^a^	0 (0)	100 (26.5%)	29 (24.6%)	0.91 [0.56;1.45]	1
Family history of dementia^a^	4 (0.8%)	100 (26.6%)	24 (20.7%)	0.72 [0.43;1.18]	1
History of cardiovascular disease^a^	0 (0)	70 (18.5%)	24 (20.3%)	1.13 [0.66;1.87]	1
History of arterial hypertension^a^	0 (0)	157 (41.5%)	54 (45.8%)	1.19 [0.78;1.80]	1
History of diabetes (type not specified)^a^	0 (0)	42 (11.1%)	10 (8.5%)	0.75 [0.34;1.49]	1
History of hypercholesterolemia^a^	0 (0)	161 (42.6%)	39 (33.1%)	0.67 [0.43;1.03]	1
Age at assessment (years)	0 (0)	67.2 (11.2)	67.5 (9.9)	1.00 [0.98;1.02]	1
Age at onset (years)	0 (0)	63.6 (11.6)	59.7 (11.2)	0.97 [0.95;0.99]	0.019*
Disease duration since diagnosis (years)	0 (0)	3.6 (3.9)	7.8 (5.4)	1.21 [1.15;1.27]	<0.001*
Hoehn and Yahr scale	0 (0)	2.0 (0.7)	2.6 (0.8)	2.74 [2.01;3.72]	<0.001*
MDS-UPDRS I	12 (2.4%)	8.9 (6.4)	14.3 (7.5)	1.11 [1.08;1.14]	<0.001*
MDS-UPDRS II	11 (2.2%)	8.8 (6.7)	18.3 (9.1)	1.16 [1.12;1.19]	<0.001*
MDS-UPDRS III	7 (1.4%)	31.4 (14.1)	44.3 (18.1)	1.05 [1.04;1.07]	<0.001*
MDS-UPDRS IV	4 (0.8%)	0.9 (2.4)	4.0 (4.6)	1.28 [1.20;1.37]	<0.001*
LEDD (g/day)	13 (2.6%)	0.4 (0.33)	0.8 (0.5)	10.6 [5.86;19.0]	<0.001*
MoCA	12 (2.4%)	24.9 (4.0)	23.8 (4.5)	0.94 [0.90;0.99]	0.369
Sniffin’ sticks score	0 (0)	8.0 (3.6)	6.9 (3.4)	0.92 [0.86;0.97]	0.036*
SCOPA-AUT	27 (5.4%)	13.1 (7.5)	18.2 (8.3)	1.08 [1.05;1.11]	<0.001*
BDI-I	23 (4.6%)	8.9 (6.7)	12.5 (8.00)	1.07 [1.04;1.10]	0.001*
PDSS	32 (6.5%)	110 (22.8)	94.7 (27.0)	0.98 [0.97;0.98]	<0.001*
RBDSQ	33 (6.7%)	3.9 (2.9)	5.5 (3.5)	1.16 [1.09;1.25]	0.001*
pRBD^a^	33 (6.7%)	85 (23.7%)	48 (45.7%)	2.70 [1.71;4.26]	0.006*
PDQ-39	41 (8.3%)	32.2 (23.3)	57.4 (28.4)	1.04 [1.03;1.05]	<0.001*
Gait disorder^a^	0 (0)	178 (47.1%)	94 (79.7%)	4.37 [2.71;7.29]	<0.001*
Falls^a^	0 (0)	36 (9.5%)	38 (32.2%)	4.49 [2.68;7.57]	<0.001*
Dyskinesia^a^	0 (0)	26 (6.9%)	32 (27.1%)	5.01 [2.84;8.93]	<0.001*
Motor fluctuations^a^	0 (0)	25 (6.6%)	51 (43.2%)	10.6 [6.22;18.6]	<0.001*
Hallucinations^a^	0 (0)	35 (9.3%)	35 (29.7%)	4.12 [2.43;7.00]	<0.001*
Impulse control disorder^a^	0 (0)	24 (6.4%)	20 (16.9%)	3.01 [1.58;5.68]	0.018*
Depression^a^	0 (0)	87 (23.0%)	32 (27.1%)	1.25 [0.77;1.99]	1
Restless legs syndrome^a^	0 (0)	25 (6.6%)	13 (11.0%)	1.76 [0.84;3.51]	1
Excessive daytime sleepiness^a^	0 (0)	94 (24.9%)	52 (44.1%)	2.38 [1.54;3.66]	0.002*
Insomnia^a^	0 (0)	88 (23.3%)	54 (45.8%)	2.77 [1.80;4.29]	<0.001*
Orthostatic hypotension^a^	0 (0)	93 (24.6%)	43 (36.4%)	1.76 [1.12;2.73]	0.363
Dysphagia^a^	0 (0)	79 (20.9%)	41 (34.7%)	2.01 [1.27;3.16]	0.072
Constipation^a^	0 (0)	150 (39.7%)	64 (54.2%)	1.80 [1.19;2.74]	0.162
Urinary incontinence^a^	0 (0)	99 (26.2%)	42 (35.6%)	1.56 [1.00;2.42]	1

*Significant *p* value after adjustment for multiple comparisons (Bonferroni).

^a^Categorical variable.

Intergroup comparisons using Student’s *t*-test (for normal distributed continuous variables), Mann–Whitney U-test (for non-normal distributed continuous variables), and the chi-square test (or Fisher’s exact test where appropriate) for categorical variables. SD, Standard deviation; OR, Odds ratio; CI, Confidence interval; MDS-UPDRS, Movement Disorder Society-Unified Parkinson’s Disease Rating Scale; H&Y, Modified Hoehn and Yahr scale; LEDD, Levodopa equivalent daily dose; MoCA, Montreal Cognitive Assessment; SCOPA-AUT, SCales for Outcomes in PArkinson’s disease-Autonomic dysfunction; BDI-I, Beck Depression Inventory-version 1; RBDSQ, Rapid Eye Movement Disorder (RBD) Screening Questionnaire; PDQ-39, PD Questionnaire-Quality of Life.

**Table 2:** Sociodemographic, clinical, and neuropsychological profile of idiopathic Parkinson’s disease (iPD) patients with or without freezing of gait (FOG).

		iPD^FOG−^	iPD^FOG+^	iPD^FOG+^ vs. iPD^FOG−^	
Detailed neuropsychological profile	Missing	Mean (SD) or YES (%)	Mean (SD) or YES (%)	OR [CI 95%]	*p* value
	*n* (%)				
Number of individuals (*n*)		126	35	-	-
MoCA	1 (0.6%)	25.1 (3.3)	24.7 (3.8)	0.96 [0.86;1.07]	1
CERAD: total score	8 (5.0%)	21.2 (4.3)	20.5 (5.5)	0.96 [0.88;1.05]	1
CERAD: delayed recall (number correct)	8 (5.0%)	6.4 (2.1)	5.9 (3.0)	0.90 [0.75;1.08]	1
CERAD: word recognition (Yes+No)	8 (5.0%)	19.3 (1.0)	18.6 (1.9)	0.69 [0.52;0.93]	1
Digit span forwards	6 (3.7%)	8.6 (2.0)	8.6 (1.9)	0.98 [0.80;1.20]	1
Digit span backwards	6 (3.7%)	5.9 (1.9)	5.6 (2.0)	0.91 [0.73;1.13]	1
FAB	1 (0.6%)	14.5 (3.0)	14.5 (3.1)	1.00 [0.88;1.13]	1
Phonemic fluency: F	2 (1.2%)	8.9 (4.3)	9.7 (4.7)	1.04 [0.96;1.13]	1
Phonemic fluency: S	5 (3.1%)	11.5 (4.5)	11.1 (4.3)	0.98 [0.90;1.07]	1
Category fluency test (number of words)	6 (3.7%)	28.4 (8.6)	30.5 (9.6)	1.03 [0.98;1.08]	1
TMT-A	3 (1.9%)	49.3 (23.5)	53.6 (17.6)	1.01 [0.99;1.02]	1
TMT-B	4 (2.5%)	118 (69.0)	149 (80.7)	1.01 [1.00;1.01]	1
(TMT-B) − (TMT-A)	4 (2.5%)	69.1 (53.0)	95.9 (69.7)	1.01 [1.00;1.01]	1
Stroop interference score	17 (10.6%)	68.8 (58.1)	76.9 (48.6)	1.00 [1.00;1.01]	1
Stroop: word reading time (s)	13 (8.1%)	51.4 (10.5)	57.0 (25.9)	1.02 [1.00;1.05]	1
Stroop: word reading errors	15 (9.3%)	0.3 (0.9)	0.6 (1.4)	1.29 [0.92;1.79]	1
Benton’s judgment of line orientation	3 (1.9%)	23.6 (4.9)	17.5 (11.3)	0.90 [0.86;0.95]	0.128
Issac’s set test	10 (6.2%)	32.6 (6.7)	32.9 (7.4)	1.01 [0.95;1.07]	0.874

*Significant *p* value after adjustment for multiple comparisons (Bonferroni).

^a^Categorical variable.

Intergroup comparisons using Student’s *t*-test (for normal distributed continuous variables), Mann–Whitney U-test (for non-normal distributed continuous variables), and the chi-square test (or Fisher’s exact test where appropriate) for categorical variables. SD, Standard deviation; OR, Odds ratio; CI, Confidence interval; MoCA, Montreal Cognitive Assessment; CERAD, Consortium to Establish a Registry for Alzheimer’s Disease; CERAD, Total score = Trial 1 + 2 + 3; FAB, Frontal assessment battery; TMT, Trail making test.

### Statistical analysis

The *compareGroups* R package was used for the univariate analyses (Subirana et al., 2014). Counts, percentages, means, and standard deviations (SDs) were reported for categorical and continuous variables in the dichotomized groups iPD^FOG+^ and iPD^FOG−^. In the comparison between iPD^FOG+^ and iPD^FOG−^, odds ratios (ORs), 95% confidence intervals (CI), and *p* values were obtained for each of the clinical and neuropsychological outcomes employing Student’s *t*-test (for normal distributed continuous variables), Mann–Whitney *U*-test (for non-normal distributed continuous variables), and the chi-square test or Fisher’s exact test for categorical variables, respectively. A prediction model using logistic regression was applied for clinical and neuropsychological variables (“tidyverse”) R package (Wickham et al., 2019). The regression analyses for iPD^FOG+^ vs. iPD^FOG−^ on clinical variables were adjusted for sex, age at assessment (AAA), Movement Disorder Society-Unified Parkinson’s Disease Rating Scale Score Part III (MDS-UPDRS III), and levodopa equivalent daily dose (LEDD). By contrast, for the subgroup analysis of neuropsychological profiles, the regression analysis was adjusted for AAA, sex, MDS-UPDRS III, LEDD, and total languages spoken. For all models, we accounted for multiple comparisons using the Bonferroni correction.

## Results

In total, 496 genotyped patients with iPD 118 showing FOG (iPD^FOG+^) and 378 without FOG (iPD^FOG−^) were included in the overall analysis. As shown in Table 1, the age at onset (AAO) was significantly lower in iPD^FOG+^ individuals (59.7 ± 11.2 vs. 63.6 ± 11.6 years, *p =* 0.019) with longer disease duration since diagnosis (7.8 ± 5.4 vs. 3.6 ± 3.9 years, *p* < 0.001) than iPD^FOG−^ individuals. Male sex was more represented in individuals with iPD^FOG+^ than in individuals with iPD^FOG−^ (74.6% vs. 63.8%), but this difference was not statistically significant after correction for multiple testing (*p* = 0.868).

**Figure 3:**
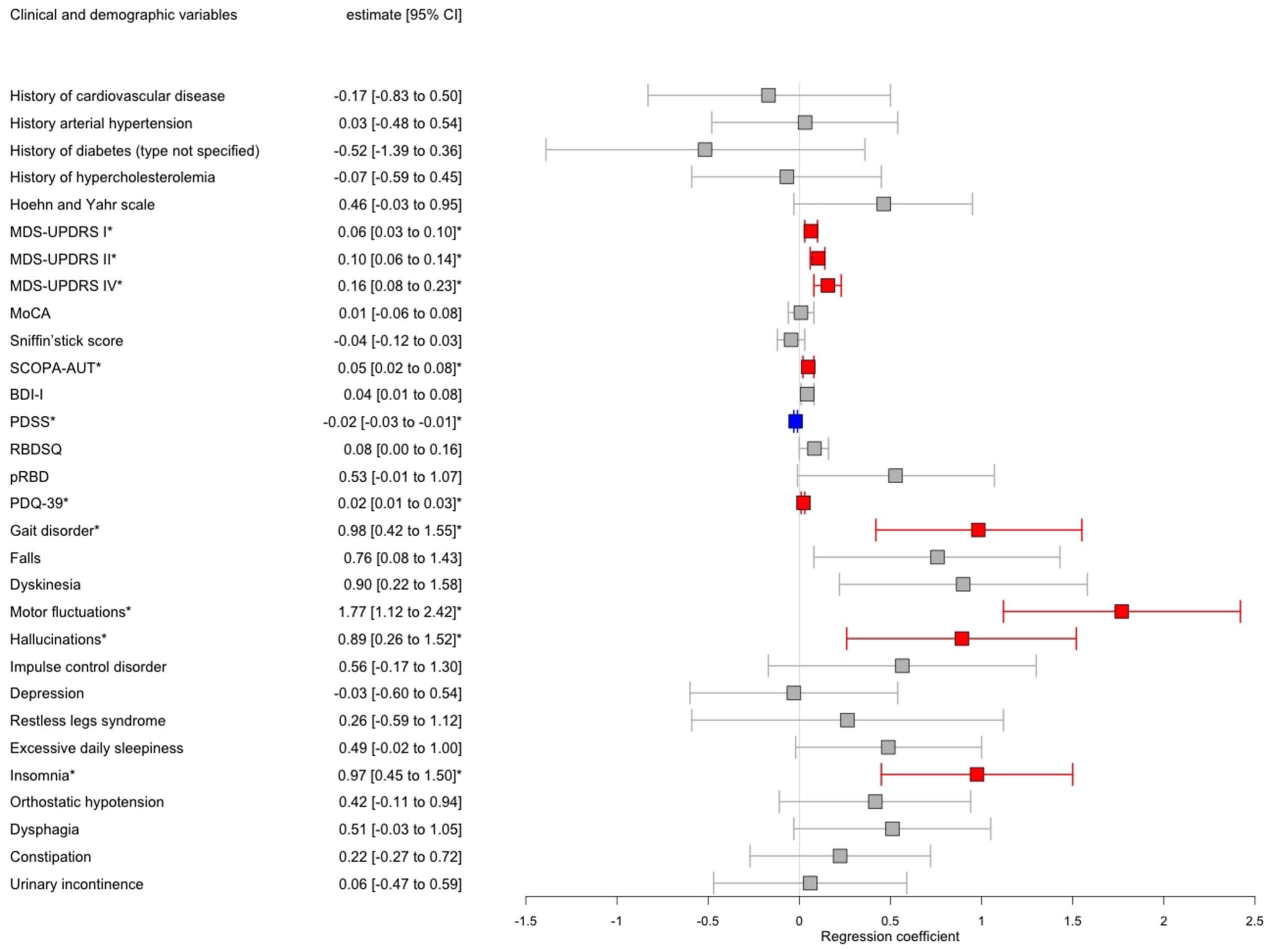
Forest plot showing results of multiple logistic regression models of patients with idiopathic Parkinson’s disease (iPD) with freezing of gait (FOG) vs. iPD without FOG adjusted for age, sex, MDS-UPDRS III, and LEDD. Significant associations after Bonferroni correction for multiple testing were annotated by an asterisk where red color indicates a positive significant association and blue color negative significant association, respectively, between iPD^FOG+^ vs. iPD^FOG−^ and the clinical variable. CI, Confidence interval; MDS-UPDRS, Movement Disorder Society-Unified Parkinson’s Disease Rating Scale; LEDD, Levodopa equivalent daily dose; MoCA, Montreal Cognitive Assessment; SCOPA-AUT, SCales for Outcomes in PArkinson’s disease-Autonomic dysfunction; BDI-I, Beck Depression Inventory-version 1; RBDSQ, Rapid Eye Movement Disorder (RBD) Screening Questionnaire; and PDQ-39, PD Questionnaire-Quality of Life.

Logistic regressions adjusted for sex, AAA, MDS-UPDRS III, and LEDD revealed significantly higher motor complications in iPD^FOG+^ vs. iPD^FOG−^ individuals (see forest plot in Figure 3), that is, a higher frequency of gait disorder (79.7% vs. 47.1%, *p* = 0.003), MDS-UPDRS II and IV (18.3 ± 9.1 vs. 8.8 ± 6.6, *p* < 0.001; 4.0 ± 4.6 vs. 0.9 ± 2.4, respectively, *p* < 0.001), and a significantly higher frequency of motor fluctuations (43.2% vs. 6.61%, *p* < 0.001). Equally, the non-motor symptoms were significantly higher in iPD^FOG+^ vs. iPD^FOG−^ individuals when assessed using MDS-UPDRS I (14.3 ± 7.5 vs. 8.9 ± 6.4, *p* = 0.006), with more pronounced autonomic dysfunction (SCOPA-AUT: 18.2 ± 8.31 vs. 13.1 ± 7.5, *p* = 0.02) and a higher frequency of hallucinations (29.7% vs. 9.3%, *p* = 0.03). Furthermore, iPD^FOG+^ individuals showed significantly lower quality of sleep (PDSS, 94.7 ± 27.0 vs. 110 ± 22.8, *p* = 0.001), with a higher frequency of reported insomnia than in iPD^FOG−^ individuals (45.8% vs. 23.3%, *p* = 0.001). Equally, the self-reported quality of life by patients measured via PDQ-39 was significantly lower in iPD^FOG+^ individuals (the higher the score in PDQ-39, the lower the quality of life: 57.4 ± 28.4 vs. 32.2 ± 23.3, *p* < 0.001).

### Cognitive subdomain analysis in iPD^FOG+^ vs. iPD^FOG−^

**Figure 4:**
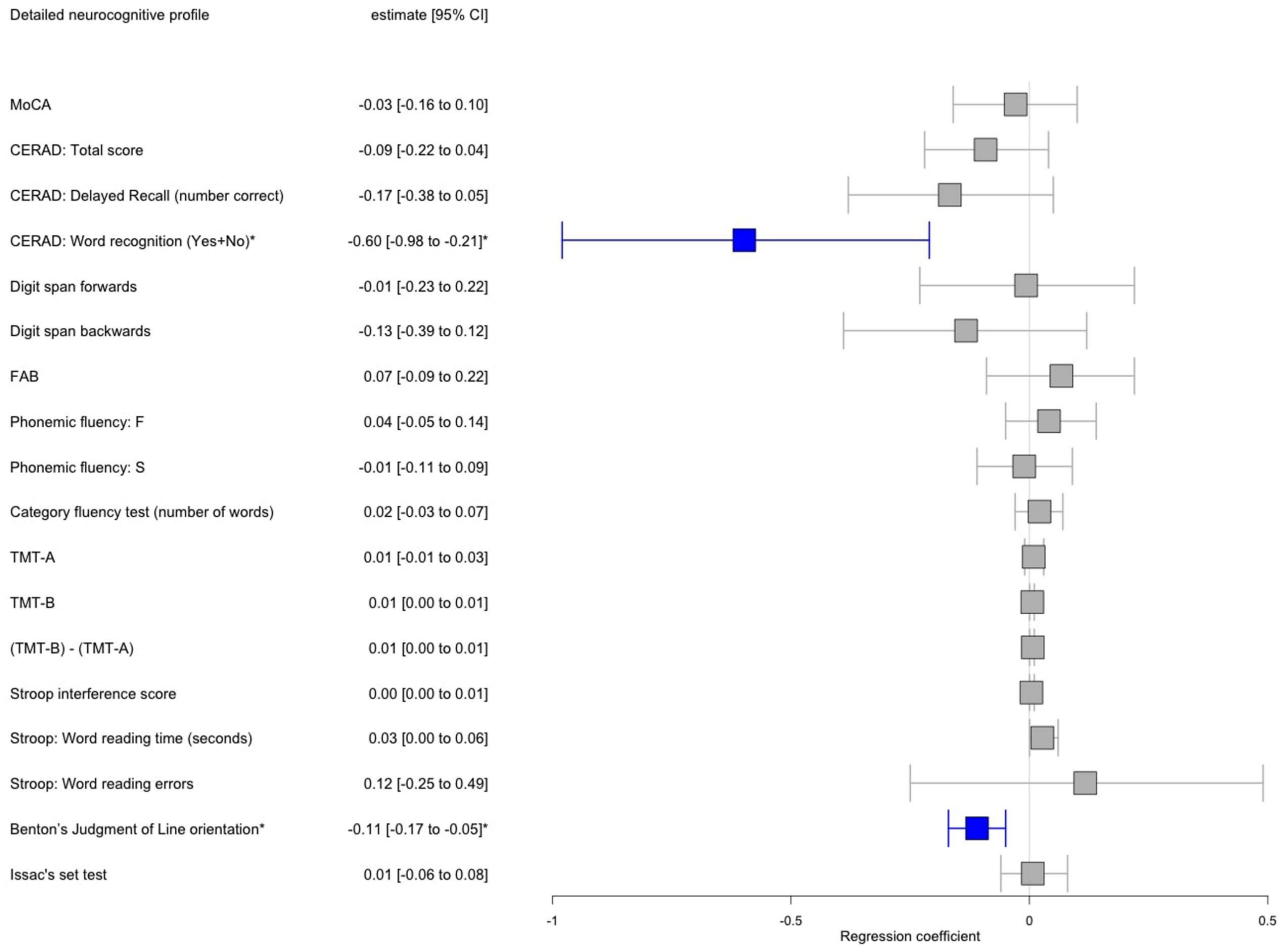
Forest plot showing results of multiple logistic regression adjusted for age, sex, MDS-UPDRS III, LEDD, and total languages spoken performed in a subset of patients with idiopathic Parkinson’s disease (iPD) with freezing of gait (FOG) vs. iPD without FOG with available detailed neuropsychological testing. Significant associations after Bonferroni correction for multiple testing were annotated by an asterisk where red color indicates a positive significant association and blue color negative significant association, respectively, between iPD^FOG+^ vs. iPD^FOG−^ and the neuropsychological outcome. MDS-UPDRS, Movement Disorder Society-Unified Parkinson’s Disease Rating Scale; LEDD, Levodopa equivalent daily dose; CI, Confidence interval; MoCA, Montreal Cognitive Assessment; CERAD, Consortium to Establish a Registry for Alzheimer’s Disease; CERAD, total score = Trial 1 + 2 + 3; FAB, Frontal assessment battery; TMT, Trail making test.

From the initial sample of 496 genotyped iPD patients, 161 patients [35 out of 118 iPD^FOG+^ (30%) and 126 out of 378 iPD^FOG−^ (33%)] underwent a detailed neuropsychological assessment. The overall descriptive statistics of the patient subgroup, including sociodemographic information, comorbidities, and PD-related scales and symptoms, are appended in Supplementary Table S1. Table 2 lists the results of neuropsychological tests used for the assessment of iPD^FOG+^ and iPD^FOG-^, with illustrations of the respective cognitive subdomains addressed in Figure 2. After adjusting for confounding effects of AAA, sex, MDS-UDPRS III, LEDD, and total languages spoken, the logistic regressions of iPD^FOG+^ vs. iPD^FOG−^ identified significantly lower scores in Benton’s Judgment of Line Orientation (17.5 ± 11.3 vs. 23.6 ± 4.9, *p* < 0.001) and Consortium to Establish a Registry for Alzheimer’s Disease (CERAD) word recognition (18.6 ± 1.91 vs. 19.3 ± 1.0, *p* = 0.01), indicating higher impairment in visuospatial domain, executive dysfunction, and memory encoding in iPD^FOG+^ (see Figure 4).

## Discussion

This study represents a large cross-sectional analysis using baseline visits of PD patients recruited from the deep-phenotyped monocentric, observational, longitudinal Luxembourg Parkinson’s study. Importantly, the neuropsychological assessment battery applied in our study comprehensively covered overall cognitive performance and all cognitive subdomains, as shown in Figure 2. In this study, we focussed on testing the hypothesis that the overall cognitive impairment or impairment in cognitive subdomains (i.e., executive dysfunction) might be linked to the development of the FOG phenomenon in PD as proposed in *the cognitive model* of FOG.

While systematically reviewed in detail elsewhere (Monaghan et al., 2023), most of the previous studies focusing on FOG and cognition were largely based on inadequate intergroup matching or adjustment for disease severity as the main determinant of FOG (Morris et al., 2020), potentially biased self-reported FOG classification (Gao et al., 2020), or low sample sizes (Heremans et al., 2013). After addressing all the limitations above, we determined a comparative cognitive performance assessed using Montreal Cognitive Assessment (MoCA) in our regression model of iPD^FOG+^ when compared to iPD^FOG−^ (23.8 ± 4.5 vs. 24.9 ± 4.0, *p* = 0.37). Such observations were not in line with a recent systematic meta-analysis demonstrating a significant pooled effect of FOG on worse cognition (*n* = 139 studies meta-analyzed for overall cognitive performance in PD with and without FOG); however, the variability of the studies included was very high with potentially large confounding effects of age, sex, and disease severity or dopaminergic medication (>50% of included meta-analyzed studies did not report a significantly higher overall cognitive impairment in PD^FOG+^ than in PD^FOG−^) (Monaghan et al., 2023). We argue that the comparable overall cognitive performance between the two groups in our study was an important setting for a cognitive subdomain analysis. The *a priori* uneven distribution of overall cognitive impairment between the investigated groups may have predetermined the dysfunction in the cognitive subdomains, potentially biasing the analysis in previous studies.

Our observation that FOG^+^ and overall cognitive impairment (MoCA) were not significantly associated in regression models might suggest that these two symptoms could be independent consequences of the disease progression as frequency and severity of cognitive impairment and FOG increase with disease progression. This hypothesis is further supported by a recent study using positron emission tomography (PET) imaging (Bohnen et al., 2019), where the common denominator for FOG and cognitive impairment in addition to neurodegeneration in the dopaminergic system was identified in a pronounced *acetylcholine* deficit in bilateral striatum, temporal, and mesiofrontal limbic regions—a deficit that occurs typically later in the disease trajectory.

On the level of cognitive subdomains, we identified a significant association between higher visuospatial impairment, executive dysfunction, and deficit in memory encoding as tested using Benton’s Judgment of Line Orientation and CERAD word recognition (Figure 4). The isolated significant difference in the CERAD word recognition task in PD^FOG+^ (vs. not significant CERAD delayed recall or CERAD total score) suggests an impairment in memory encoding mainly caused by executive dysfunction. This finding corroborated the *cognitive model* of FOG, which posits that the mechanism underlying FOG is rooted in conflict-resolution deficits as a consequence of executive dysfunction (Nieuwboer and Giladi, 2013). Furthermore, our results corroborated multiple previous studies replicating the association of FOG with executive dysfunction in cross-sectional and longitudinal settings (Peterson et al., 2016; Amboni et al., 2010; Vandenbossche et al., 2011). In our study, the visuospatial orientation was found highly impaired in PD^FOG+^ in comparison with PD^FOG−^, pointing to a more widespread cortical deficit beyond the executive subdomain, further supported by the observation of more frequent hallucinations in the PD^FOG+^ group. Of note, a cross-comparison to previous studies investigating a subdomain cognitive function must be taken with caution due to the vast variability of the neuropsychological assessment employed for each cognitive subdomain and various (or even lacking) matching strategies between the investigated groups.

Although our models were adjusted for the confounding effect of overall PD medication (LEDD) and motor severity quantified using MDS-UPDRS III, we determined a higher rate of motor fluctuations in PD^FOG+^ vs. PD^FOG−^ (quantitatively using MDS-UPDRS IV and qualitatively by frequency of motor fluctuations). Equally, FOG was associated with a non-motor dominant endophenotype in our study with a higher frequency of hallucinations and autonomic dysfunction and a lower quality of sleep. Importantly, the non-motor dominant endophenotype was reconfirmed when disease duration (instead of MDS-UPDRS III and LEDD) was used as a covariate in regression models to match disease severity (appended in Supplementary Figure S1). Our finding of a non-motor dominant endophenotype with high motor complications in PD strongly corresponded to a fast-progressing subtype of PD with more axial symptoms recently identified and cross-validated in a longitudinal study of more than 1,000 patients using three independent cohorts [Parkinson’s Progression Marker Initiative (PPMI), Luxembourg Parkinson’s study, and ICEBERG study] and a data-driven approach (Hähnel et al., 2024).

Of note, we fitted all the models in an idiopathic setting after excluding all carriers of PD-related mutations. The lack of genotyping in previous studies constituted an important limitation, given that several highly prevalent pathological gene variants in PD (such as in the *GBA1* gene) are linked to more severe phenotypes, including higher cognitive impairment or dementia (all pathogenic variants and the distribution of *GBA1* carriers among the groups in our study before exclusion were appended in Supplementary material) (Pachchek et al., 2023). In addition, we considered the disease severity to influence substantially both motor complications and non-motor symptoms (including cognition) and hence adjusted all regression models for sociodemographic confounders and the disease severity using MDS-UPDRS III and LEDD.

Additional strengths of the presented study included (i) relatively large sample sizes per group, (ii) clinical determination of FOG by investigators during a face-to-face visit to avoid a misclassification by the self-reported questionnaires, and (iii) excluding neurological comorbidities potentially confounding the FOG (stroke) or cognition (brain tumor, history of encephalitis, comorbid normal pressure hydrocephalus, or epilepsy). Nevertheless, this study had its limitations. Due to the cross-sectional setup of the study, we could not establish a causal effect between FOG and the identified endophenotype. In addition, we tested our study participants solely in the ON state. It was observed that not only the motor symptoms of PD but also the cognitive performance can be significantly influenced by dopaminergic medication and treatment state (ON vs. OFF state) (Lewis and Barker, 2009; Chaudhuri and Schapira, 2009). However, we accounted partially for such confounding treatment effect by including LEDD as a covariate in the regression models. Finally, since deep neuropsychological profiling was an optional assessment in our study, we cannot completely exclude an inherent participation bias in our data collection.

In summary, our study revealed more widespread cortical dysfunction associated with FOG in PD beyond the proposed executive dysfunction in the frame of the cognitive model of FOG. Furthermore, the significant association between FOG and dominantly non-motor endophenotype in PD should be well-considered in the clinical setting in terms of treatment adaptations and in the design of future clinical trials.

## NCER-PD consortium

Geeta Acharya, Luxembourg Institute of Health, Strassen, Luxembourg; Gloria Aguayo, Luxembourg Institute of Health, Strassen, Luxembourg; Myriam Alexandre, Luxembourg Institute of Health, Strassen, Luxembourg; Muhammad Ali, Luxembourg Center for Systems Biomedicine, University of Luxembourg, Esch-sur-Alzette, Luxembourg; Wim Ammerlann, Luxembourg Institute of Health, Strassen, Luxembourg; Rudi Balling, Luxembourg Center for Systems Biomedicine, University of Luxembourg, Esch-sur-Alzette, Luxembourg; Michele Bassis, Luxembourg Center for Systems Biomedicine, University of Luxembourg, Esch-sur-Alzette, Luxembourg; Katy Beaumont, Luxembourg Institute of Health, Strassen, Luxembourg; Regina Becker, Luxembourg Center for Systems Biomedicine, University of Luxembourg, Esch-sur-Alzette, Luxembourg; Camille Bellora, Luxembourg Institute of Health, Strassen, Luxembourg; Guy Berchem, Center Hospitalier de Luxembourg, Strassen, Luxembourg; Daniela Berg, Center of Neurology and Hertie Institute for Clinical Brain Research, Department of Neurodegenerative Diseases, University Hospital Tübingen, Tübingen, Germany; Alexandre Bisdorff, Center Hospitalier Emile Mayrisch, Esch-sur-Alzette, Luxembourg; Kathrin Brockmann, Center of Neurology and Hertie Institute for Clinical Brain Research, Department of Neurodegenerative Diseases, University Hospital Tübingen, Tübingen, Germany; Jessica Calmes, Luxembourg Institute of Health, Strassen, Luxembourg; Lorieza Castillo, Luxembourg Institute of Health, Strassen, Luxembourg; Gessica Contesotto, Luxembourg Institute of Health, Strassen, Luxembourg; Giuseppe Arena, Luxembourg Center for Systems Biomedicine, University of Luxembourg, Esch-sur-Alzette, Luxembourg; Nico Diederich, Center Hospitalier de Luxembourg, Strassen, Luxembourg; Rene Dondelinger, Center Hospitalier Emile Mayrisch, Esch-sur-Alzette, Luxembourg; Daniela Esteves, Luxembourg Institute of Health, Strassen, Luxembourg; Guy Fagherazzi, Luxembourg Institute of Health, Strassen, Luxembourg; Jean-Yves Ferrand, Luxembourg Institute of Health, Strassen, Luxembourg; Manon Gantenbein, Luxembourg Institute of Health, Strassen, Luxembourg; Thomas Gasser, Center of Neurology and Hertie Institute for Clinical Brain Research, Department of Neurodegenerative Diseases, University Hospital Tübingen, Tübingen, Germany; Piotr Gawron, Luxembourg Center for Systems Biomedicine, University of Luxembourg, Esch-sur-Alzette, Luxembourg; Soumyabrata Ghosh, Luxembourg Center for Systems Biomedicine, University of Luxembourg, Esch-sur-Alzette, Luxembourg; Marijus Giraitis, Luxembourg Institute of Health, Strassen, Luxembourg; Center Hospitalier de Luxembourg, Strassen, Luxembourg; Enrico Glaab, Luxembourg Center for Systems Biomedicine, University of Luxembourg, Esch-sur-Alzette, Luxembourg; Clarissa Gomes, Luxembourg Center for Systems Biomedicine, University of Luxembourg, Esch-sur-Alzette, Luxembourg; Elisa Gómez De Lope, Luxembourg Center for Systems Biomedicine, University of Luxembourg, Esch-sur-Alzette, Luxembourg; Jérôme Graas, Luxembourg Institute of Health, Strassen, Luxembourg; Mariella Graziano, Association of Physiotherapists in Parkinson’s Disease Europe, Esch-sur-Alzette, Luxembourg; Valentin Groues, Luxembourg Center for Systems Biomedicine, University of Luxembourg, Esch-sur-Alzette, Luxembourg; Anne Grünewald, Luxembourg Center for Systems Biomedicine, University of Luxembourg, Esch-sur-Alzette, Luxembourg; Wei Gu, Luxembourg Center for Systems Biomedicine, University of Luxembourg, Esch-sur-Alzette, Luxembourg; Gaël Hammot, Luxembourg Institute of Health, Strassen, Luxembourg; Anne-Marie Hanff, Luxembourg Institute of Health, Strassen, Luxembourg; Linda Hansen, Luxembourg Center for Systems Biomedicine, University of Luxembourg, Esch-sur-Alzette, Luxembourg; Center Hospitalier de Luxembourg, Strassen, Luxembourg; Maxime Hansen, Luxembourg Center for Systems Biomedicine, University of Luxembourg, Esch-sur-Alzette, Luxembourg; Center Hospitalier de Luxembourg, Strassen, Luxembourg; Michael Heneka, Luxembourg Center for Systems Biomedicine, University of Luxembourg, Esch-sur-Alzette, Luxembourg; Estelle Henry, Luxembourg Institute of Health, Strassen, Luxembourg; Sylvia Herbrink, Center Hospitalier du Nord, Ettelbrück, Luxembourg; Sascha Herzinger, Luxembourg Center for Systems Biomedicine, University of Luxembourg, Esch-sur-Alzette, Luxembourg; Michael Heymann, Luxembourg Institute of Health, Strassen, Luxembourg; Michele Hu, Oxford Parkinson’s Disease Center, Nuffield Department of Clinical Neurosciences, University of Oxford, Oxford, United Kingdom; Alexander Hundt, Luxembourg Institute of Health, Strassen, Luxembourg; Ivana Paccoud, Luxembourg Institute of Health, Strassen, Luxembourg; Nadine Jacoby, Private practice, Ettelbruck, Luxembourg; Jacek Jaroslaw Lebioda, Luxembourg Center for Systems Biomedicine, University of Luxembourg, Esch-sur-Alzette, Luxembourg; Yohan Jaroz, Luxembourg Center for Systems Biomedicine, University of Luxembourg, Esch-sur-Alzette, Luxembourg; Quentin Klopfenstein, Luxembourg Center for Systems Biomedicine, University of Luxembourg, Esch-sur-Alzette, Luxembourg; Jochen Klucken, Luxembourg Center for Systems Biomedicine, University of Luxembourg, Esch-sur-Alzette, Luxembourg; Luxembourg Institute of Health, Strassen, Luxembourg; Center Hospitalier de Luxembourg, Strassen, Luxembourg; Rejko Krüger, Luxembourg Center for Systems Biomedicine, University of Luxembourg, Esch-sur-Alzette, Luxembourg; Luxembourg Institute of Health, Strassen, Luxembourg; Center Hospitalier de Luxembourg, Strassen, Luxembourg; Pauline Lambert, Luxembourg Institute of Health, Strassen, Luxembourg; Zied Landoulsi, Luxembourg Center for Systems Biomedicine, University of Luxembourg, Esch-sur-Alzette, Luxembourg; Roseline Lentz, Parkinson Luxembourg Association, Leudelange, Luxembourg; Inga Liepelt, Center of Neurology and Hertie Institute for Clinical Brain Research, Department of Neurodegenerative Diseases, University Hospital Tübingen, Tübingen, Germany; Robert Liszka, Westpfalz-Klinikum GmbH, Kaiserslautern, Germany; Laura Longhino, Center Hospitalier de Luxembourg, Strassen, Luxembourg; Victoria Lorentz, Luxembourg Institute of Health, Strassen, Luxembourg; Paula Cristina Lupu, Luxembourg Institute of Health, Strassen, Luxembourg; Clare Mackay, Oxford Center for Human Brain Activity, Wellcome Center for Integrative Neuroimaging, Department of Psychiatry, University of Oxford, Oxford, United Kingdom; Walter Maetzler, Department of Neurology, University Medical Center Schleswig-Holstein, Kiel, Germany; Katrin Marcus, Ruhr-University of Bochum, Bochum, Germany; Guilherme Marques, Luxembourg Institute of Health, Strassen, Luxembourg; Tainá Marques, Luxembourg Center for Systems Biomedicine, University of Luxembourg, Esch-sur-Alzette, Luxembourg; Patricia Martins Conde, Luxembourg Center for Systems Biomedicine, University of Luxembourg, Esch-sur-Alzette, Luxembourg; Patrick May, Luxembourg Center for Systems Biomedicine, University of Luxembourg, Esch-sur-Alzette, Luxembourg; Deborah Mcintyre, Luxembourg Institute of Health, Strassen, Luxembourg; Chouaib Mediouni, Luxembourg Institute of Health, Strassen, Luxembourg; Francoise Meisch, Luxembourg Center for Systems Biomedicine, University of Luxembourg, Esch-sur-Alzette, Luxembourg; Myriam Menster, Luxembourg Institute of Health, Strassen, Luxembourg; Maura Minelli, Luxembourg Institute of Health, Strassen, Luxembourg; Michel Mittelbronn, Luxembourg Center for Systems Biomedicine, University of Luxembourg, Esch-sur-Alzette, Luxembourg; Laboratoire National de Santé, Dudelange, Luxembourg; Brit Mollenhauer, Paracelsus-Elena-Klinik, Kassel, Germany; Carlos Moreno, Luxembourg Center for Systems Biomedicine, University of Luxembourg, Esch-sur-Alzette, Luxembourg; Friedrich Mühlschlegel, Laboratoire National de Santé, Dudelange, Luxembourg; Romain Nati, Center Hospitalier de Luxembourg, Strassen, Luxembourg; Ulf Nehrbass, Luxembourg Institute of Health, Strassen, Luxembourg; Sarah Nickels, Luxembourg Center for Systems Biomedicine, University of Luxembourg, Esch-sur-Alzette, Luxembourg; Beatrice Nicolai, Center Hospitalier de Luxembourg, Strassen, Luxembourg; Jean-Paul Nicolay, Private practice, Luxembourg, Luxembourg; Fozia Noor, Luxembourg Institute of Health, Strassen, Luxembourg; Marek Ostaszewski, Luxembourg Center for Systems Biomedicine, University of Luxembourg, Esch-sur-Alzette, Luxembourg; Sinthuja Paccontrolshek, Luxembourg Center for Systems Biomedicine, University of Luxembourg, Esch-sur-Alzette, Luxembourg; Claire Pauly, Luxembourg Center for Systems Biomedicine, University of Luxembourg, Esch-sur-Alzette, Luxembourg; Center Hospitalier de Luxembourg, Strassen, Luxembourg; Laure Pauly, Luxembourg Center for Systems Biomedicine, University of Luxembourg, Esch-sur-Alzette, Luxembourg; Lukas Pavelka, Luxembourg Center for Systems Biomedicine, University of Luxembourg, Esch-sur-Alzette, Luxembourg; Luxembourg Institute of Health, Strassen, Luxembourg; Center Hospitalier de Luxembourg, Strassen, Luxembourg; Magali Perquin, Luxembourg Institute of Health, Strassen, Luxembourg; Rosalina Ramos Lima, Luxembourg Institute of Health, Strassen, Luxembourg; Armin Rauschenberger, Luxembourg Center for Systems Biomedicine, University of Luxembourg, Esch-sur-Alzette, Luxembourg; Rajesh Rawal, Luxembourg Center for Systems Biomedicine, University of Luxembourg, Esch-sur-Alzette, Luxembourg; Dheeraj Reddy Bobbili, Luxembourg Center for Systems Biomedicine, University of Luxembourg, Esch-sur-Alzette, Luxembourg; Eduardo Rosales, Luxembourg Institute of Health, Strassen, Luxembourg; Isabel Rosety, Luxembourg Center for Systems Biomedicine, University of Luxembourg, Esch-sur-Alzette, Luxembourg; Kirsten Rump, Luxembourg Center for Systems Biomedicine, University of Luxembourg, Esch-sur-Alzette, Luxembourg; Estelle Sandt, Luxembourg Institute of Health, Strassen, Luxembourg; Stefano Sapienza, Luxembourg Center for Systems Biomedicine, University of Luxembourg, Esch-sur-Alzette, Luxembourg; Venkata Satagopam, Luxembourg Center for Systems Biomedicine, University of Luxembourg, Esch-sur-Alzette, Luxembourg; Margaux Schmitt, Luxembourg Institute of Health, Strassen, Luxembourg; Sabine Schmitz, Luxembourg Center for Systems Biomedicine, University of Luxembourg, Esch-sur-Alzette, Luxembourg; Reinhard Schneider, Luxembourg Center for Systems Biomedicine, University of Luxembourg, Esch-sur-Alzette, Luxembourg; Jens Schwamborn, Luxembourg Center for Systems Biomedicine, University of Luxembourg, Esch-sur-Alzette, Luxembourg; Jean-Edouard Schweitzer, Luxembourg Center for Systems Biomedicine, University of Luxembourg, Esch-sur-Alzette, Luxembourg; Amir Sharify, Luxembourg Institute of Health, Strassen, Luxembourg; Ekaterina Soboleva, Luxembourg Center for Systems Biomedicine, University of Luxembourg, Esch-sur-Alzette, Luxembourg; Kate Sokolowska, Luxembourg Institute of Health, Strassen, Luxembourg; Olivier Terwindt, Luxembourg Center for Systems Biomedicine, University of Luxembourg, Esch-sur-Alzette, Luxembourg; Center Hospitalier de Luxembourg, Strassen, Luxembourg; Hermann Thien, Luxembourg Institute of Health, Strassen, Luxembourg; Elodie Thiry, Center Hospitalier de Luxembourg, Strassen, Luxembourg; Rebecca Ting Jiin Loo, Luxembourg Center for Systems Biomedicine, University of Luxembourg, Esch-sur-Alzette, Luxembourg; Christophe Trefois, Luxembourg Center for Systems Biomedicine, University of Luxembourg, Esch-sur-Alzette, Luxembourg; Johanna Trouet, Luxembourg Institute of Health, Strassen, Luxembourg; Olena Tsurkalenko, Luxembourg Institute of Health, Strassen, Luxembourg; Michel Vaillant, Luxembourg Institute of Health, Strassen, Luxembourg; Mesele Valenti, Luxembourg Institute of Health, Strassen, Luxembourg; Sijmen Van Schagen, Luxembourg Center for Systems Biomedicine, University of Luxembourg, Esch-sur-Alzette, Luxembourg; Liliana Vilas Boas, Center Hospitalier de Luxembourg, Strassen, Luxembourg; Maharshi Vyas, Luxembourg Center for Systems Biomedicine, University of Luxembourg, Esch-sur-Alzette, Luxembourg; Richard Wade-Martins, Oxford Parkinson’s Disease Center, Department of Physiology, Anatomy and Genetics, University of Oxford, Oxford, United Kingdom; Paul Wilmes, Luxembourg Center for Systems Biomedicine, University of Luxembourg, Esch-sur-Alzette, Luxembourg; Evi Wollscheid-Lengeling, Luxembourg Center for Systems Biomedicine, University of Luxembourg, Esch-sur-Alzette, Luxembourg; Gelani Zelimkhanov, Center Hospitalier de Luxembourg, Strassen, Luxembourg. Department of Neurology Philipps, University Marburg, Marburg, Germany.

## Data Availability

The code for this study can be found at: https://doi.org/10.17881/ymfh-0z15. The dataset presented in this study is not publicly available as it is linked to the Luxembourg Parkinson’s study and its internal regulations. Any reasonable requests for accessing the dataset can be directed to: request.ncer-pd@uni.lu.
