# Supplementary material for: Genetically stratified Parkinson’s disease with freezing of gait is related to specific pattern of cognitive impairment and non-motor dominant endophenotype
Source: Front Aging Neurosci. 2024 Oct 11;16:1479572. doi: 10.3389/fnagi.2024.1479572 (PMC11502444; doi:10.3389/fnagi.2024.1479572)
Supplement: Supplementary file 1 [file Table_1.DOCX]

Supplementary material

**Table S1**. Sociodemographic and clinical characteristics of sub-group analysis of idiopathic Parkinson’s disease (iPD) patients with or without freezing of gait (FOG) that underwent a detailed neurocognitive profiling.

|  |  |  |  |  |
| --- | --- | --- | --- | --- |
|  | **iPD^FOG-^** | **iPD^FOG+^** | **iPD^FOG+^ vs. iPD^FOG-^** |  |
| **Clinical and demographic variables** | Mean (SD) or % YES | Mean (SD) or % YES | OR [CI 95%] | *p*-value |
| Number of individuals (n) | 126 | 35 | - | - |
| Age at assessment (years) | 65.9 (10.8) | 65.3 (9.8) | 0.99 [0.96;1.03] | 1 |
| Sex (male) ^a^ | 85 (67.5%) | 26 (74.3%) | 1.38 [0.61;3.39] | 1 |
| Years of education | 13.5 (3.7) | 12.7 (4.0) | 0.94 [0.85;1.04] | 1 |
| Total languages spoken | 3.2 (0.9) | 2.57 (1.2) | 0.56 [0.39;0.81] | 0,261 |
| Disease duration since diagnosis (years) | 3.5 (4.2) | 6.5 (4.8) | 1.15 [1.05;1.25] | 0,047 |
| H&Y | 2.0 (0.6) | 2.2 (0.5) | 1.92 [1.05;3.51] | 0,699 |
| MDS-UPDRS I | 9.0 (6.4) | 11.2 (6.3) | 1.05 [0.99;1.11] | 1 |
| MDS-UPDRS II | 8.5 (5.9) | 14.2 (7.5) | 1.13 [1.07;1.20] | 0,006* |
| MDS-UPDRS III | 29.2 (14.4) | 35.6 (15.5) | 1.03 [1.00;1.05] | 1 |
| MDS-UPDRS IV | 1.2 (2.7) | 4.5 (4.6) | 1.27 [1.14;1.41] | 0,009* |
| Sniffin’ stick score | 8.7 (3.3) | 8.4 (2.7) | 0.97 [0.86;1.09] | 1 |
| PDQ-39 | 32.3 (22.0) | 48.9 (24.1) | 1.03 [1.01;1.05] | 0,022* |
| SPARK | 13.8 (5.4) | 12.9 (5.2) | 0.97 [0.90;1.04] | 1 |
| SCOPA-AUT | 13.4 (7.7) | 16.1 (7.3) | 1.05 [1.00;1.10] | 1 |
| PDSS | 109 (23.0) | 93.6 (29.6) | 0.98 [0.96;0.99] | 0,277 |
| RBDSQ | 4.4 (3.2) | 4.7 (3.2) | 1.04 [0.92;1.17] | 1 |
| BDI-I | 9.4 (6.9) | 10.7 (7.9) | 1.02 [0.97;1.08] | 1 |
| LEDD (gram/day) | 0.4 (0.3) | 0.8 (0.5) | 8.06 [2.85;22.8] | 0,023* |
| Family history of parkinsonism ^a^ | 39 (31.0%) | 12 (34.3%) | 1.17 [0.51;2.57] | 1 |
| Family history of dementia ^a^ | 35 (28.0%) | 6 (17.1%) | 0.54 [0.19;1.35] | 1 |
| pRBD^a^ | 36 (29.3%) | 12 (35.3%) | 1.32 [0.57;2.94] | 1 |
| History of cardiovascular disease ^a^ | 28 (22.2%) | 5 (14.3%) | 0.60 [0.19;1.59] | 1 |
| History of arterial hypertension ^a^ | 54 (42.9%) | 14 (40.0%) | 0.89 [0.41;1.91] | 1 |
| History of diabetes (type not specified) ^a^ | 15 (11.9%) | 3 (8.6%) | 0.72 [0.15;2.39] | 1 |
| History of hypercholesterolemia ^a^ | 53 (42.1%) | 13 (37.1%) | 0.82 [0.37;1.76] | 1 |
| Gait disorder ^a^ | 59 (46.8%) | 24 (68.6%) | 2.45 [1.12;5.64] | 0,813 |
| Falls ^a^ | 12 (9.5%) | 7 (20.0%) | 2.38 [0.81;6.57] | 1 |
| Dyskinesia ^a^ | 8 (6.4%) | 12 (34.3%) | 7.51 [2.77;21.5] | 0,002* |
| Motor fluctuations ^a^ | 7 (5.6%) | 11 (31.4%) | 7.59 [2.69;23.0] | 0,003* |
| Hallucinations ^a^ | 11 (8.7%) | 7 (20.0%) | 2.61 [0.88;7.36] | 1 |
| ICD ^a^ | 9 (7.1%) | 6 (17.1%) | 2.69 [0.82;8.21] | 1 |
| Depression ^a^ | 29 (23.0%) | 8 (22.9%) | 1.00 [0.39;2.38] | 1 |
| RLS ^a^ | 12 (9.5%) | 1 (2.9%) | 0.32 [0.01;1.71] | 1 |
| Excessive daytime sleepiness ^a^ | 25 (19.8%) | 7 (20.0%) | 1.02 [0.37;2.53] | 1 |
| Insomnia ^a^ | 23 (18.3%) | 17 (48.6%) | 4.18 [1.86;9.47] | 0,012* |
| Orthostatic hypotension ^a^ | 27 (21.4%) | 10 (28.6%) | 1.47 [0.60;3.40] | 1 |
| Dysphagia ^a^ | 18 (14.3%) | 13 (37.1%) | 3.51 [1.48;8.28] | 0,115 |
| Constipation ^a^ | 40 (31.7%) | 14 (40.0%) | 1.43 [0.65;3.11] | 1 |
| Urinary incontinence ^a^ | 28 (22.2%) | 9 (25.7%) | 1.22 [0.49;2.85] | 1 |
|  |  |  |  |  |
|  |  |  |  |  |

* Significant *p-*value after adjustment for multiple comparisons (Bonferroni).

^a^ Categorical variable

Intergroup comparisons using Student’s t-test (for normal distributed continuous variables), Mann Whitney *U*-test (for non-normal distributed continuous variables) and chi-square test (or Fisher’s exact test where appropriate) for categorical variables respectively. *Abbreviations:* SD (standard deviation); OR (odds ratio); CI (confidence interval) MDS-UPDRS (Movement Disorder Society- Unified Parkinson’s Disease Rating Scale); H&Y (modified Hoehn and Yahr scale); LEDD (Levodopa Equivalent Daily Dose); MoCA (Montreal Cognitive Assessment); SCOPA-AUT (SCales for Outcomes in PArkinson’s disease- Autonomic dysfunction); BDI-I (Beck Depression Inventory- version 1); RBDSQ (Rapid Eye Movement Disorder (RBD) Screening Questionnaire); PDQ-39 (PD questionnaire -Quality of Life).

**
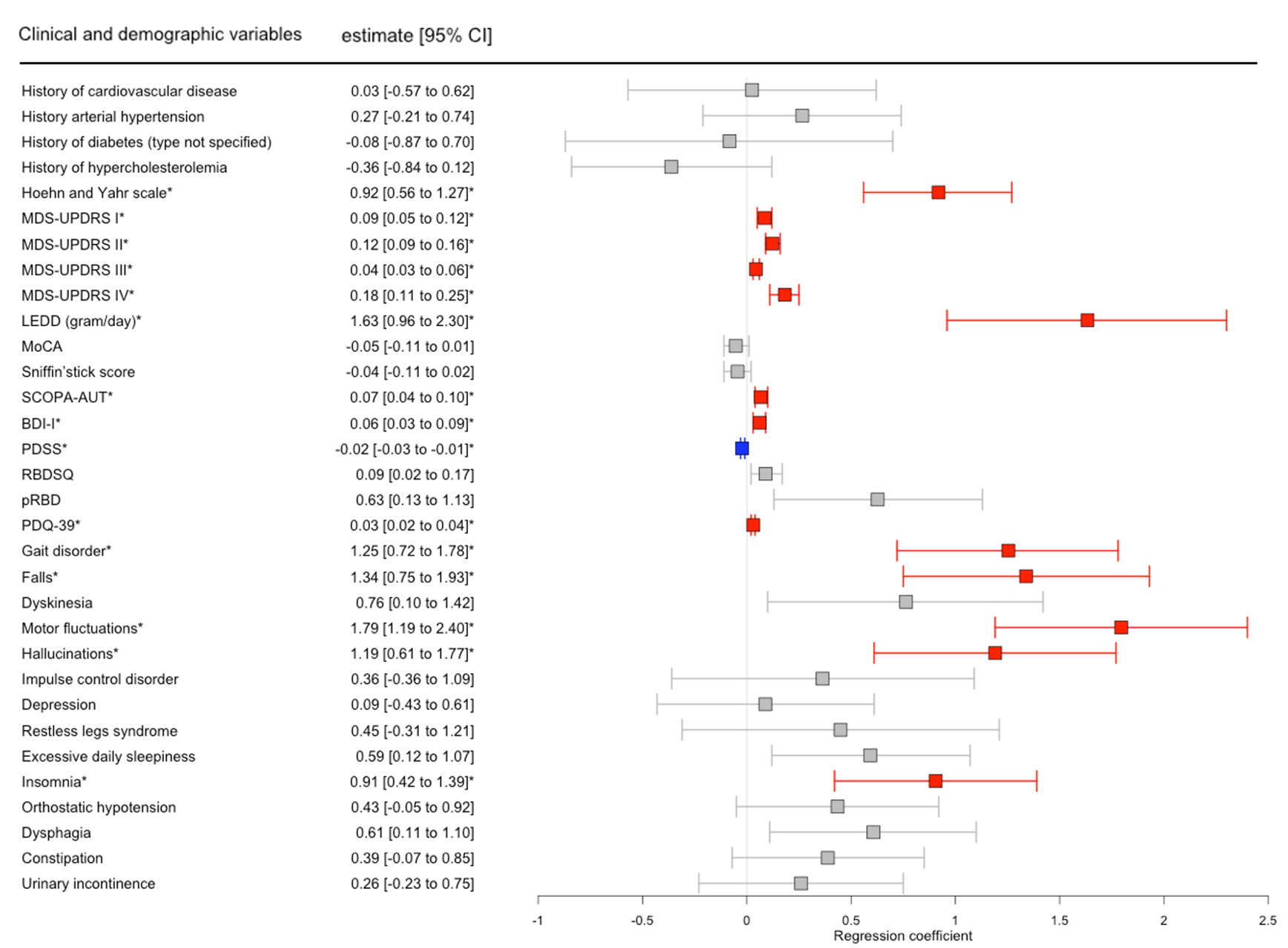
Figure S1.** Forest plot showing results of multiple logistic regression model of patients with idiopathic Parkinson’s disease (iPD) with freezing of gait (FOG) vs. iPD without FOG adjusted for age, *disease duration* and sex. Significant associations after Bonferroni correction for multiple testing were annotated by an asterisk where red colour indicates positive significant association and blue colour negative significant association, respectively, between iPD^FOG+^ vs. iPD^FOG-^ and the clinical variable. *Abbreviations:* CI (confidence interval) MDS-UPDRS (Movement Disorder Society- Unified Parkinson’s Disease Rating Scale); LEDD (Levodopa Equivalent Daily Dose); MoCA (Montreal Cognitive Assessment); SCOPA-AUT (SCales for Outcomes in PArkinson’s disease- Autonomic dysfunction); BDI-I (Beck Depression Inventory- version 1); RBDSQ (Rapid Eye Movement Disorder (RBD) Screening Questionnaire); PDQ-39 (PD questionnaire -Quality of Life).

**PD causing rare variants were defined by the ClinVar classification as “pathogenic/likely pathogenic:**

- Patients with Parkinson's disease (PD) excluded from overall analysis of PD endophenotype to FOG due to carrier status of PD-linked mutation:

GBA1 (heterozygot, p.A215D); GBA1 (heterozygot, p.E365K); GBA1 (heterozygot, p.G241R); GBA1 (heterozygot, p.L483P); GBA1 (heterozygot, p.P161S); GBA1 (heterozygot, p.R398X); GBA1 (heterozygot, p.T408M); GBA1 (heterozygot, p.G234W); GBA1 (heterozygot, p.N409S); LRRK2 (heterozygot, p.G2019S), copy number variation (CNV) in PRKN.

Proportion of mutation carriers in GBA1 in overall analysis of PD endophenotype to FOG:

17 PD ^GBA1+/FOG+^ out of 146 PD^FOG+^ = 11.6%

49 PD ^GBA1+/FOG-^ out of 425 PD^FOG-^ = 11.5%

- Patients with PD excluded from cognitive subdomain analysis of PD due to carrier status of PD-linked mutation:

GBA1 (heterozygot, c.115+1G>A); GBA1 (heterozygot, p.A215D); GBA1 (heterozygot; p.E365K); GBA1 (heterozygot, p.G241R); GBA1 (heterozygot, p.L483P); GBA1 (heterozygot, p.N409S); GBA1 (heterozygot, p.P161S); GBA1 (heterozygot; p.R398X); GBA1 (heterozygot; p.T408M); GBA1 (heterozygot, p.G234W); GBA1 (heterozygot, p.R502H); GBA1 (homozygot; p.E365K); GBA1 (heterozygot, p.N409S); GBA1 (heterozygot; p.L483P); LRRK2 (heterozygot, p.G2019S) LRRK2; (heterozygot, p.R1441C); LRRK2 (heterozygot, p.R1441S); LRRK2 (heterozygot, p.G2019S); PINK1 (homozygot, p.L369P); CNV in PRKN.

Proportion of mutation carriers in GBA1 in the cognitive subdomain analysis of PD:

8 PD^GBA1+/FOG+^ out of 59 PD^FOG+^ = 13.6%

17 PD ^GBA1+/FOG-^ out of 172 PD^FOG-^ = 9.9%
